# Comparison of subcostal transversus abdominis block with intraperitoneal instillation of bupivacaine and dexmedetomidine for pain relief after laparoscopic cholecystectomy, randomized double blinded controlled study

**DOI:** 10.1186/s12871-025-03412-4

**Published:** 2025-11-14

**Authors:** Ahmed Ismail Abd El Sabour, Hamdy Abbas Youssef, Eman Ahmed Ismail, Dina Hatem Ahmed

**Affiliations:** 1 Surgical Intensive Care and Pain management, Faculty of Medicine, New Valley University, Kharga, Egypt; 2 Surgical Intensive Care and Pain management, Faculty of Medicine, Assiut University, Assiut, Egypt; 3 Department of Anesthesia, Intensive Care & Pain Management, New Valley University, kharga, Egypt

**Keywords:** Subcostal transversus abdominis block, Intraperitoneal instillation, Dexmedetomidine, Laparoscopic cholecystectomy

## Abstract

**Background:**

This study compared the analgesic effectiveness of ultrasound-guided subcostal transversus abdominis plane (STAP) block with intraperitoneal instillation of bupivacaine and dexmedetomidine after LC.

**Methods:**

This prospective, randomized, double-blind trial involved 60 ASA I–II patients undergoing elective LC. At random, the patients were divided into two groups of thirty each. Group 1 was given STAP block with bupivacaine and dexmedetomidine along with intraperitoneal normal saline. Bupivacaine, dexmedetomidine was administered intraperitoneally to Group 2 along with STAP with normal saline. The Numerical Rating Scale (NRS) was used to measure postoperative pain at rest, while coughing, and for shoulder tip discomfort as a primary outcome. Patient satisfaction, sedation rating, and time to first analgesic demand were also assessed as a secondary outcome.

**Results:**

At rest and while coughing, Group 1’s NRS ratings were considerably lower at all times (*p* < 0.001). Group 1 experienced considerably less shoulder tip discomfort 24 h after surgery (*p* = 0.032). Group 1 had a longer time to first analgesic demand (16.5 vs. 11.7 h, *p* = 0.003). By the end of the research, 12 patients in Group 1 needed postoperative analgesia (46.2% vs. 96.4%, *p* < 0.001). Group 1 had increased patient satisfaction (*p* < 0.001).

**Conclusion:**

STAP block offers better postoperative analgesia than intraperitoneal instillation.

**Trial registration:**

This prospective interventional double-blinded clinical trial was done at Assiut University Hospitals, Assiut, Egypt, From June, 2021 to May, 2024. The study protocol was approved by the Institutional Review Board of the Faculty of Medicine, Assiut University (IRB approval number: 17200580) and registered with ClinicalTrials.gov (ID: NCT04715165).

## Introduction

For the surgical treatment of biliary lithiasis, laparoscopic cholecystectomy (LC) has almost supplanted open cholecystectomy as the gold standard because of its minimally invasive nature [1]. Even with its benefits, postoperative pain management is still a major problem in LC, affecting hospital time, morbidity, perioperative stress levels, and patient recovery [2, 3].

There are several reasons for the complicated character of post-LC pain. The typical shoulder tip discomfort is caused by diaphragmatic irritation, while the visceral component results from tissue damage in the anterior abdominal wall during trocar insertion [4].

In recent years, regional block-based techniques have gained popularity in multimodal pain management. An advancement over the conventional TAP block is the ultrasound-guided (USG) subcostal transversus abdominis plane (STAP) block, which was initially reported by Hebbard in 2008 [5]. Preliminary studies have demonstrated that STAP block is superior to port-site infiltration, conventional opioid analgesia, and routine TAP block in LC patients [6–8].

At the same time, local anesthetics administered intraperitoneally (IP) have become a viable alternate method of analgesia. This method focuses on diaphragmatic irritation-induced transferred shoulder pain (C3, C4) and visceral pain sources [9]. In the early postoperative phase after LC, IP injection of local anesthetics demonstrated promise in lowering pain intensity ratings, which might enhance recovery profiles [10].

Adjuvants have been investigated to improve and extend the analgesic effects of local anesthetics. The α2-adrenergic receptor agonist as dexmedetomidine when used in conjunction with local anesthetics has been shown to be effective in extending both sensory and motor blockade [11]. Because of its high lipophilia, it absorbs quickly, which may extend and improve the quality of analgesia.

This study aimed to compare STAP block and intraperitoneal instillation efficacy using bupivacaine and dexmedetomidine in controlling postoperative pain following LC.

### Ethics approval and consent to participate

A prospective, randomized, double-blind trial was conducted from June 2021 to May 2024. The study protocol was filed with ClinicalTrials.gov (ID: NCT04715165) and approved by the Faculty of Medicine’s Institutional Review Board at Assiut University (IRB approval number: 17200580). The Helsinki Declaration’s criteria were followed in the conduct of the study. All patients provided written informed consent once the purpose of the trial was explained.

### Patients and methods

#### Patients

The study comprised sixty ASA I or II patients, aged 20 to 65, who were scheduled for elective laparoscopic cholecystectomy under general anesthesia. Exclusion criteria included patient refusal, pregnancy, body mass index more than 35 kg/m2, inability to understand the research protocol, and contraindications to study drugs or regional anesthetics.

#### Randomization

The participants were split up into two groups of thirty patients each using a computer-generated random number table. Only after patients were enrolled were the opaque, sealed envelopes containing group allocations unsealed. Neither the doctor or nurse (investigator) nor the participant (patient) were aware of patient allocation or drug used. The study drugs were prepared by one of the anesthesia supervisors (not included in the procedure, observation or data collection).

## Methods

Each patient underwent a consistent general anesthesia protocol, entered the anesthetic room, had an intravenous access set up, and underwent routine monitoring, which included electrocardiography, non-invasive blood pressure, heart rate, pulse oximetry, and capnogram.

After pre-oxygenation, fentanyl (2 µg/kg) and propofol (1.5–2.5 mg/kg) were used to induce anesthesia. Cis-atracurium (0.15 mg/kg) was administered to ease tracheal intubation. To keep the end-tidal carbon dioxide tension between 35-40mmHg, oxygen-assisted mechanical ventilation was started. Cis-atracurium (0.03 mg/kg), oxygen, and isoflurane were used to maintain the anesthetic. Lactated Ringer’s solution was given at a rate of 6–8 mL/kg to provide intraoperative fluid control.

All patients got intravenous paracetamol (15 mg/kg) after surgery. Atropine (0.02 mg/kg) and neostigmine (0.05 mg/kg) restored neuromuscular blockade. After extubating and full recovery, the patients were sent to the post-anesthesia care unit (PACU).

Intervention Protocols Group 1 (USG-STAP Block): Bilateral USG-STAP blocks were carried out after anesthesia was administered. A 100-mm, 22G block needle was guided to the neurovascular plane between the rectus abdominis and transversus abdominis muscles using a high-frequency linear probe that was placed beneath the xiphisternum and moved laterally along the subcostal border to the anterior axillary line. Dexmedetomidine vial is 200 mic in 2 ml, the vial was diluted to 20 ml volume with normal saline so that each 1 ml contains 10 mic of dexmedetomidine, the volume of dexmedetomidine was withdrawn according to the patient weight, then 10 ml of bupivacaine 0.5% was added, lastly the volume is then completed to 20 ml volume with normal saline.

After negative aspiration, Above mixture was injected 20 ml into each side. A 40 mL intraperitoneal injection of normal saline was administered at the end of the operation.

Group 2 (Intraperitoneal Instillation): At the conclusion of the procedure, just before the trocar was removed, the surgeon directly injected above mixture at the gallbladder bed and under both diaphragmatic domes. Following the onset of anesthesia, the USG-STAP block was performed using normal saline.

The numerical rating pain scale (NRS), which is evaluated from 0 to 10 (0 being no pain and 10 being the greatest possible pain), was the main result [12, 13]. The quality of analgesia during coughing and at rest was assessed by a PACU nurse who was not aware of the research design at 10, 30, 2, 4, 8, 12, 16, and 24 h following the conclusion of operation. NRS also assessed the level of shoulder tip pain over these time periods. When the pain score was between 3 and 6/10, paracetamol 15 mg/kg was recommended as an analgesic; however, when the pain score was more than 6/10, more opioid analgesics (fentanyl 1mic/kg) were required.

The number of patients who required rescue analgesia during the first 24 h and the duration until the initial analgesic need were recorded are examples of secondary outcomes.

Patient satisfaction was assessed by Likert score [14]:

1 = Excellent, 2 = Good, 3 = Fairly well, 4 = poor.

Side effects include local anesthetic toxicity, technique complications and drug adverse complications (bradycardia, nausea and vomiting).

### Sample size

Based on a prior research [15], A power calculation estimated that in order to detect an effect size of 1.02 difference of mean NRS scale between the studied groups, with a p-value < 0.05 and 80% power, confidence level 0.95, a sample size of 52 patients was needed (26 for each group). This calculated using G power 3.1. In order to account for possible dropouts, the needed sample size of 26 patients per group was raised to 30.

### Statistical analysis

To analyze the data, SPSS software version 22 was utilized. The Shapiro-Wilk test was used to assess if continuous variables were normal. Frequencies were used to represent categorical data, whereas means (SD) or medians (interquartile range) were used to represent continuous and discrete variables, respectively. Fisher’s exact test or the chi-squared test were used to compare categorical data. Continuous data with normal and skewed distributions were subjected to independent sample t-tests and Mann-Whitney U tests, respectively. The time to first analgesic request was compared using the Kaplan-Meier test. Statistical significance was defined as P-values below 0.05.

## Results

**Fig. 1:**
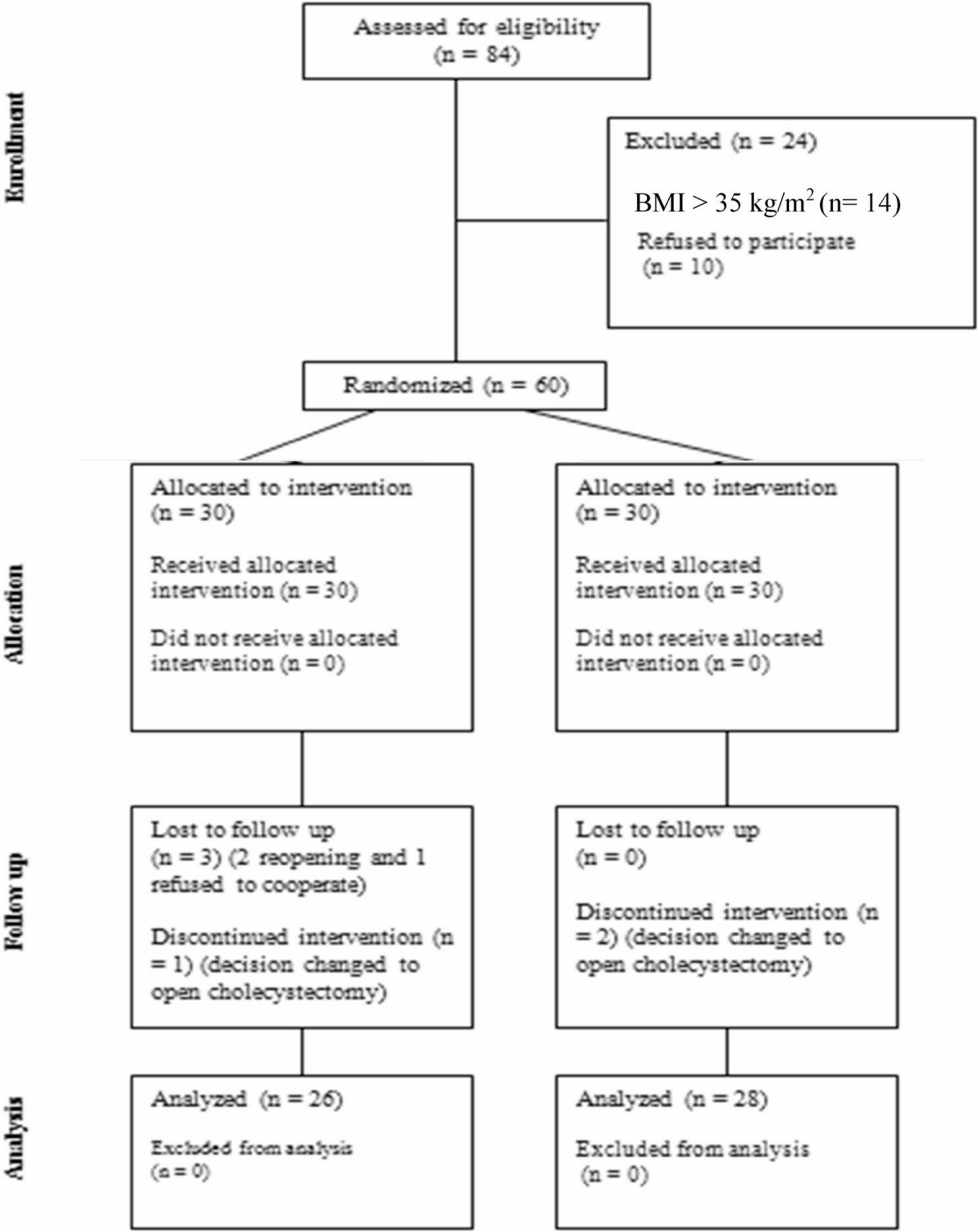
Consolidated standards of reporting trial statement flow diagram

Sixty patients were enrolled in the study divided equally in both groups. Six patients were removed from the study, three were lost to follow up (two reopened and one refused to cooperate), three for discontinued intervention (decision changed to open cholecystectomy) leaving 54 patients included. 26 from group 1 and 28 patients from group II were analyzed (Figure 1).

### Demographic data

No statistically significant differences between the studied groups regarding patients and surgical data (Table 1).

**Table 1:** Patients and surgical data

	Group I(*n* = 26)	Group II(*n* = 28)	*P*-Value
Age (years)	37.1 ± 8.6	38.5 ± 8.6	0.577
Sex (male, female)	10/16	7/21	0.251
BMI kg/m2	30.6 ± 2.9	30.4 ± 3.0	0.786
ASA (I/II)	14/12	19/9	0.153
Duration of anesthesia (min)	62.1 ± 7.6	61.8 ± 12.0	0.917
Duration of surgery (min)	44.6 ± 3.6	45.9 ± 6.1	0.36

Data are presented as mean±SD, numbers as appropriate, t-test and chi square test was done

p value <0.5 is considered significant

### Pain and analgesic outcomes

**Fig. 2:**
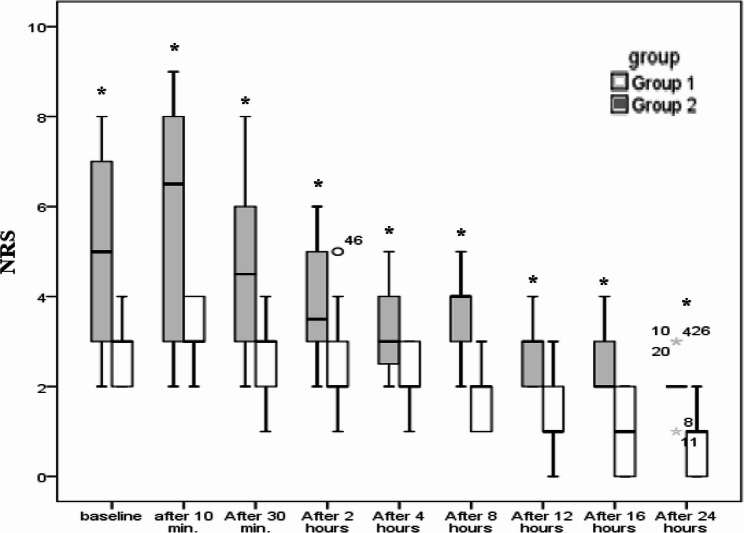
Numerical rating scale during rest

**Fig. 3:**
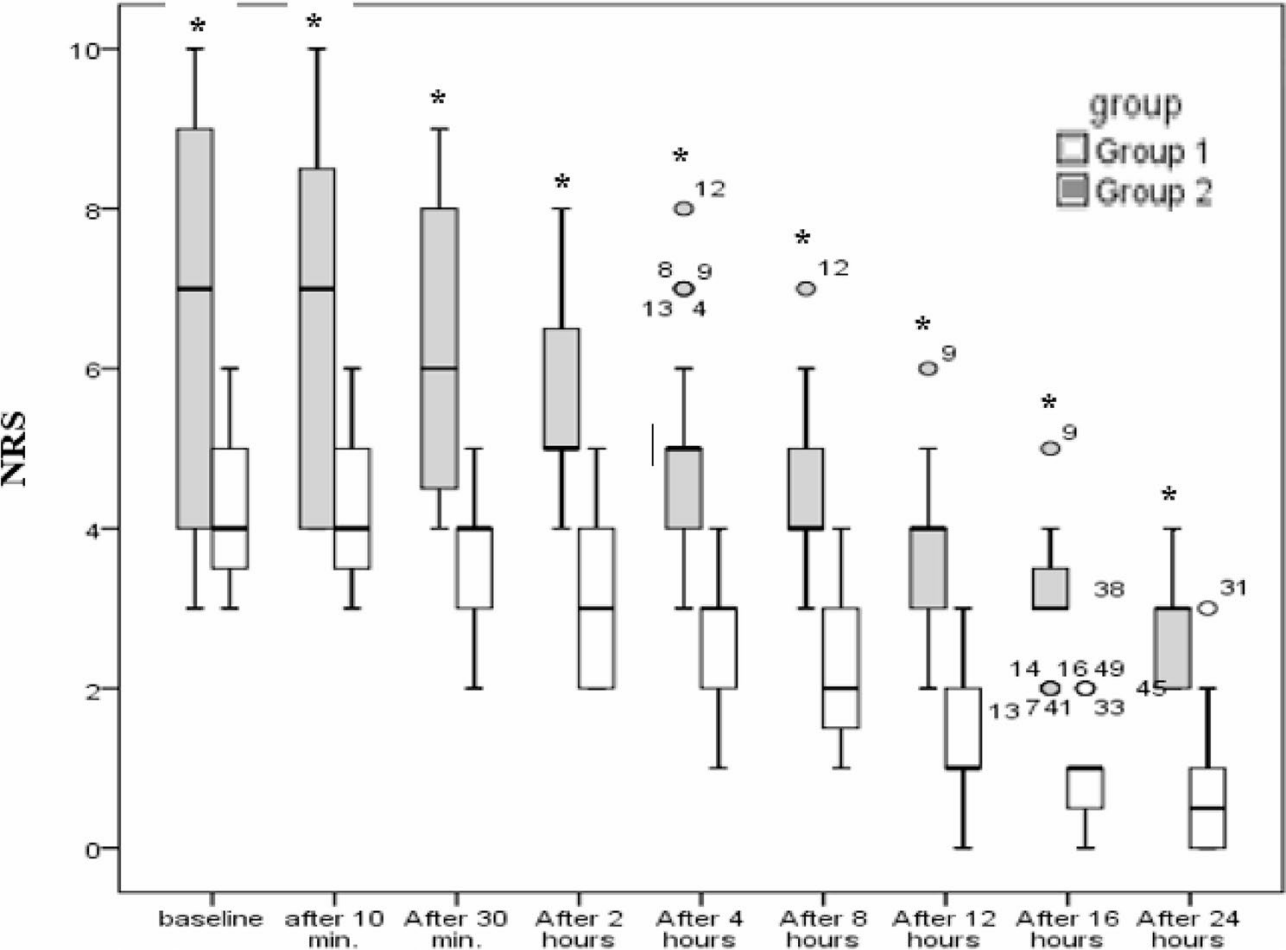
Numeric rating scale during cough

Pain scores were assessed using the NRS at multiple time points postoperatively. Group 1 consistently demonstrated lower pain scores compared to Group 2 across all time intervals. For instance, at recovery, Group 1 reported a median NRS score of 3 (2–4), while Group 2 reported a higher score of 5 (2–8) (*p* < 0.001). This trend continued up to 24 h postoperatively. These findings suggest a significantly better pain control in Group 1​(Figure 2 and Figure 3).

**Fig. 4:**
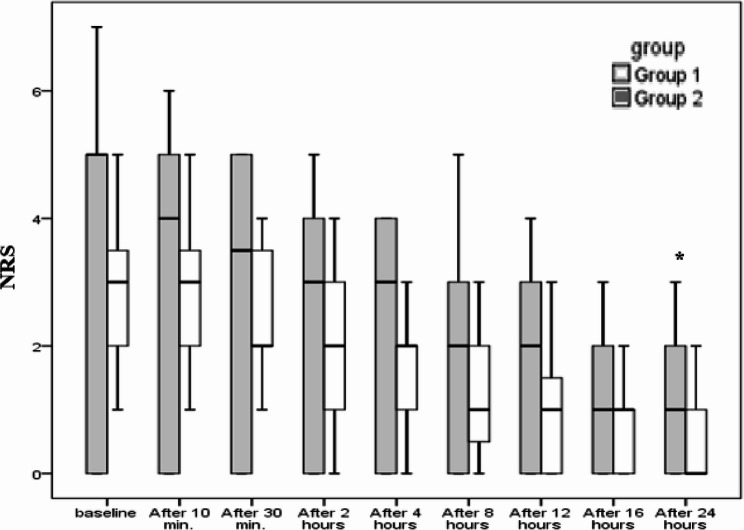
Severity of shoulder tip pain

A similar pattern was observed for the NRS during coughing, where Group 1 reported significantly lower scores compared to Group 2 throughout the postoperative period (*p* < 0.001). NRS for shoulder tip pain also showed a significant reduction in Group 1 after 24 h (*p* = 0.032), though no significant differences were observed at earlier time points ​(Figure 4).

### Analgesic requirements

**Fig. 5:**
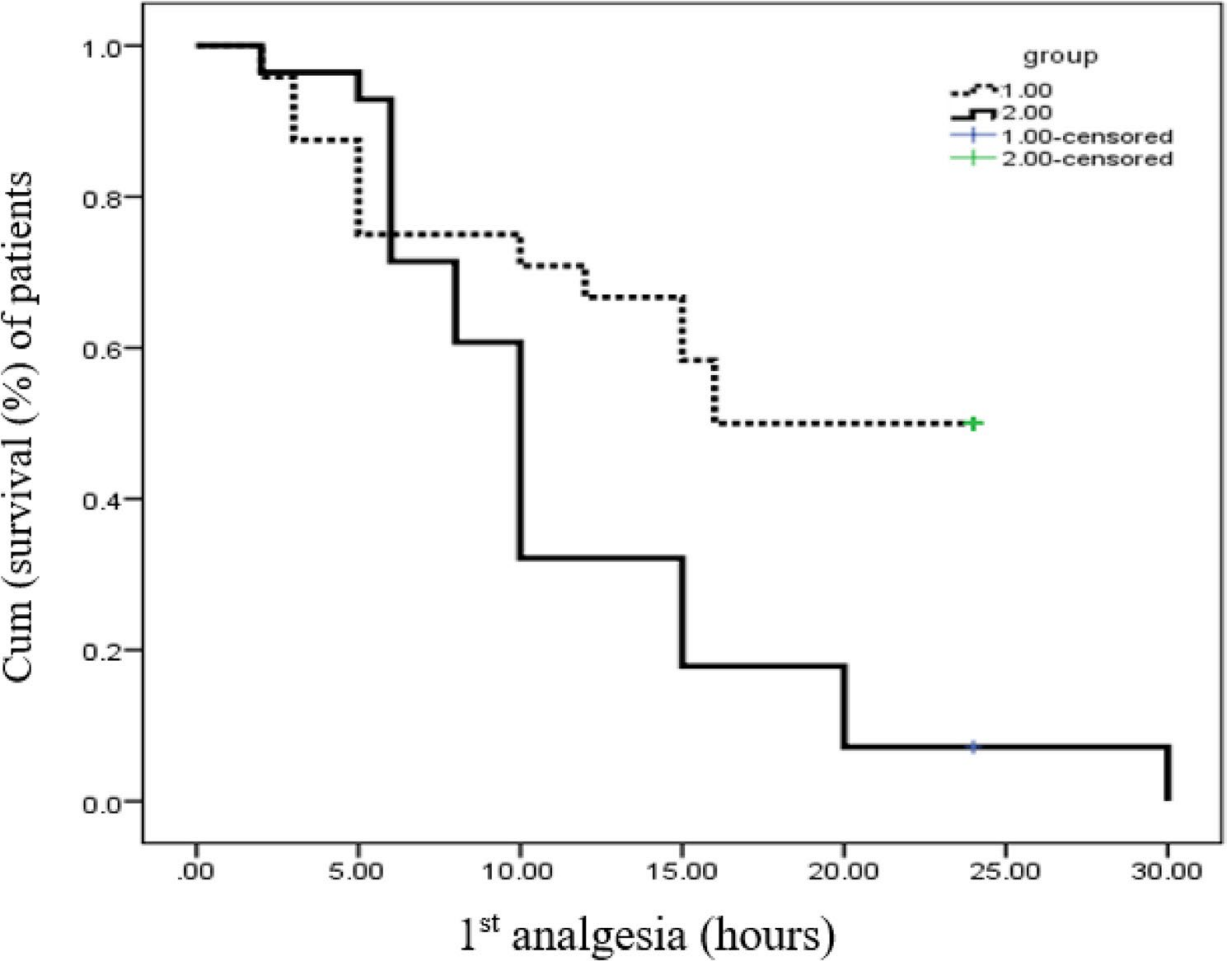
Analgesic requirement

With a mean of 16.5 h, Group 1 had a considerably longer duration before the first rescue analgesia than Group 2 (11.7 h) (*p* < 0.005). Additionally, the proportion of patients requiring postoperative analgesia was much lower in Group 1, with only 12 patients (46.2%) requiring additional analgesics, compared to 27 patients (96.4%) in Group 2 (*p* < 0.001),​ (Table 2 and Figure 5).

**Table 2:** Patient satisfaction score

	Group I(*n* = 26)	Group II(*n* = 28)	*P*-value
Excellent	13(50%)	5(18%)	0.015*
Good	4(15%)	8(28.5%)	
Fairly well	7(27%)	5(18%)	
Poor	2(8%)	10(35.5%)	

Data are presented as number(percentage), Chi square test was used to compare between both groups

p value <0.5 is considered significant

Patient satisfaction, as measured by the Likert scale, was also significantly higher in Group 1, with 50% of patients reporting excellent satisfaction compared to 18% in Group 2 with p value 0.015. This further supports the overall better postoperative experience in Group 1.

## Discussion

In patients having laparoscopic cholecystectomy (LC), this prospective, randomized, double-blind clinical trial sought to evaluate the analgesic effectiveness of intraperitoneal injections of dexmedetomidine and bupivacaine in comparison to ultrasound-guided subcostal transversus abdominis plane (STAP) block.

Our results show that, in comparison to intraperitoneal instillation, STAP block offers better postoperative analgesia.

The STAP block group’s Numerical Rating Scale (NRS) ratings for pain during rest and coughing were statistically considerably lower than those of the intraperitoneal group at all postoperative time points. This outcome is in line with studies by Khandelwal H et al. (2019), which discovered that, only for the first six hours following surgery, the TAP block group’s resting NRS scores were lower than those of the intraperitoneal group [4]. According to our research, STAP block provides an analgesic effect that lasts longer.

Interestingly, Matam R et al. (2023) found that the TAP block group had lower NRS ratings within six hours after surgery [16], which contrasts with our data. This discrepancy could have resulted from our adjuvant use of dexmedetomidine, which prolonged the analgesic effect. In *agreement to our study in 2021 Eshak MEH et* ***al.*** [17] he found that TAP block patients had significant lower pain scores at rest and after 6 h and during cough for 6 h post operative.

*In agreement to our study* In 2021 meta-analysis by *Chaw SH et al.* [18] concluded that there was significant decrease in pain scores at rest, at 2 h, also on movement at 12 h, and 24 h after the operation in the TAP block group.

According to our research, the STAP block group required fewer analgesics overall in the first 24 h after surgery and had a noticeably longer wait time to obtain an analgesic first. This is in line with research done by Khandelwal et al. (2019) [4] and Eshak MEH et al. (2021) [17].

It found that TAP block groups consumed less analgesics overall and had a delayed time to initial painkiller request compared to intraperitoneal instillation. The extended analgesia seen in our experiment could have been caused by the addition of dexmedetomidine to bupivacaine. Dexmedetomidine as an adjuvant to local anesthetics considerably reduced pain scores at numerous postoperative time points and lengthened the time to first analgesic request, according to a meta-analysis by Sun C et al. (2024), which supports this [19].

Our research suggests that the overall impact of intraperitoneal injection of local anesthetics may be less important than that of STAP block, despite the fact that it has been shown to be beneficial in reducing visceral pain components in LC. This finding contrasts with that of a research by Manan A et al. (2020), which discovered that a large dose of diluted bupivacaine greatly decreased pain [20]. Differences in the amount, technique, or concentration of the local anesthetic may be the cause of the disparity.

### Limitations

First, the research length might be extended beyond 24 h. Second, the study was constrained by the patient’s hospital discharge. The second drawback is that more patients might be used to identify the uncommon adverse effects of both methods. Finally, as the study was single-centered, multicenter participation can enhance the research.

## Conclusion

According to our research, ultrasound-guided STAP block with dexmedetomidine and bupivacaine provides superior postoperative analgesia following laparoscopic cholecystectomy compared to intraperitoneal instillation of the same medications. Reduced pain ratings, more time before seeking an analgesic, a reduction in the total quantity of analgesics used, and improved patient satisfaction without appreciably altering side effects or patient satisfaction.

Abbreviation.

## Data Availability

The data sets used and analyzed during the current study are available from the **corresponding author** upon reasonable request.

## Abbreviations

STAP: subcostal Transverses abdominus Block
LC: laparoscopic cholecystectomy
ASA: American society of Anesthesiologist
NRS: Numerical Rating Scale
USG: Ultrasound guided
IP: Intraperitoneal
IRB: Institutional Review Board
SD: standard Deviation
TAP: Transverses abdominus Block
PACU: post-anesthesia care unit
BMI: Body mass index

## References

[1] Ji W, Li L-T, Li J-S. Role of laparoscopic subtotal cholecystectomy in the treatment of complicated cholecystitis. Hepatobiliary & pancreatic diseases international: HBPD INT. 2006;5(4):584–9. 17085347

[2] Cuschieri A. Laparoscopic cholecystectomy. J R Coll Surg Edinb. 1999;44(3):187–92. 10372492

[3] Berggren U, Gordh T, Grama D, Haglund U, Rastad J, Arvidsson D. Laparoscopic versus open cholecystectomy: hospitalization, sick leave, analgesia and trauma responses. J Br Surg. 1994;81(9):1362–5. 10.1002/bjs.1800810936 7953415

[4] Khandelwal H, Parag K, Singh A, Anand N, Govil N. Comparison of subcostal transversus abdominis block with intraperitoneal instillation of Levobupivacaine for pain relief after laparoscopic cholecystectomy: a prospective study. Anesth Essays Researches. 2019;13(1):144–8. 10.4103/aer.AER_3_19 PMC6444963 31031495

[5] Hebbard P. Subcostal transversus abdominis plane block under ultrasound guidance. Anesth Analgesia. 2008;106(2):674–5. 10.1213/ane.0b013e318161a88f 18227342

[6] Siddiqui MRS, Sajid MS, Uncles DR, Cheek L, Baig MK. A meta-analysis on the clinical effectiveness of transversus abdominis plane block. J Clin Anesth. 2011;23(1):7–14. 21296242 10.1016/j.jclinane.2010.05.008

[7] Bharti N, Kumar P, Bala I, Gupta V. The efficacy of a novel approach to transversus abdominis plane block for postoperative analgesia after colorectal surgery. Anesth Analgesia. 2011;112(6):1504–8. 10.1213/ANE.0b013e3182159bf8 21467560

[8] Petersen PL, Stjernholm P, Kristiansen VB, Torup H, Hansen EG, Mitchell AU, et al. The beneficial effect of transversus abdominis plane block after laparoscopic cholecystectomy in day-case surgery: a randomized clinical trial. Anesth Analgesia. 2012;115(3):527–33. 10.1213/ANE.0b013e318261f16e 22763903

[9] Yadava A, Rajput SK, Katiyar S, Jain RK. A comparison of intraperitoneal bupivacaine-tramadol with bupivacaine-magnesium sulphate for pain relief after laparoscopic cholecystectomy: A prospective, randomised study. Indian J Anaesth. 2016;60(10):757–62. 27761040 10.4103/0019-5049.191696 PMC5064701

[10] Narchi P, Benhamou D, Fernandez H. Intraperitoneal local anaesthetic for shoulder pain after day-case laparoscopy. Lancet. 1991;338(8782–8783):1569–70. 1683981 10.1016/0140-6736(91)92384-e

[11] Coursin DB, Coursin DB, Maccioli GA, Dexmedetomidine. Curr Opin Crit Care. 2001;7(4):221–6. 11571417 10.1097/00075198-200108000-00002

[12] Jensen MP, Karoly P, Braver S. The measurement of clinical pain intensity: a comparison of six methods. Pain. 1986;27(1):117–26. 3785962 10.1016/0304-3959(86)90228-9

[13] Xia Y, Sun Y, Liu J. Effects of Dezocine on PAED scale and Ramsay sedation scores in patients undergoing NUSS procedure. Am J Translational Res. 2021;13(5):5468. PMC8205843 34150145

[14] Rosenkrantz AB, Meng X, Ream JM, Babb JS, Deng FM, Rusinek H, et al. Likert score 3 prostate lesions: association between whole-lesion ADC metrics and pathologic findings at MRI/ultrasound fusion targeted biopsy. J Magn Reson Imaging. 2016;43(2):325–32. 26131965 10.1002/jmri.24983

[15] Dr. Pratik kadam, Dr. Anjali Bhure, Dr.Sumita Bhargava. Port site infiltration versus subcostal TAP block in laparoscopic cholecystectomy for postoperative Anaglesia. Int J Med Anesthesiology. 2021;4(2):91–4.

[16] Matam R, Chawla V, Trivedi S, Bhardwaj A, Singh S. Ultrasound-Guided transversus abdominis plane block versus intraperitoneal instillation of bupivacaine after laparoscopic Cholecystectomy–A randomized control trial. J Med Sci. 2023;9(2):169.

[17] Eshak MEH, Allah SSWR, Mohamed HMAH, Esmaeil MAMAN. Comparison of ultrasound guided transversus abdominis plane block versus intraperitoneal and perioportal bupivacaine infiltration in post operative analgesia after laparoscopic cholecystectomy. QJM: Int J Med. 2021;114(Supplement1):hcab086.

[18] Chaw SH, Lo YL, Goh S-L, Cheong CC, Tan WK, Loh PS, et al. Transversus abdominis plane block versus intraperitoneal local anesthetics in bariatric surgery: a systematic review and network meta-analysis. Obes Surg. 2021;31:4305–15. 34282569 10.1007/s11695-021-05564-x

[19] Sun C, He Z, Feng B, Huang Y, Liu D, Sun Z. Effect of intraperitoneal instillation of Dexmedetomidine with local anesthetics in laparoscopic cholecystectomy: A systematic review and Meta-analysis of randomized trials. Surg Laparoscopy Endoscopy Percutaneous Techniques. 2024;34(2):222–32. 10.1097/SLE.0000000000001262 38359350

[20] Manan A, Khan AA, Ahmad I, Usman M, Jamil T, Sajid MA. Intraperitoneal bupivacaine as post-laparoscopic cholecystectomy analgesia. J Coll Physicians Surg Pak. 2020;30(1):9–12. 31931924 10.29271/jcpsp.2020.01.09

